# Supplementary material for: A new method of Bayesian causal inference in non-stationary environments
Source: PLoS One. 2020 May 22;15(5):e0233559. doi: 10.1371/journal.pone.0233559 (PMC7244155; doi:10.1371/journal.pone.0233559)
Supplement: Attachment — Submitted filename: report-fin-34250.pdf [file pone.0233559.s001.pdf]

Referee Report on Manuscript # PONE-D-19-34250

The paper "A new method of Bayesian causal inference in non-stationary environments" by Shuji Shinohara *et al.* deals with a new method of estimation of the time-varying expected value of a process. The theory is based on a classical Bayesian framework, where each new observation is used to update the estimates. Some numerical issues are discussed and a simulation study for the mean of the Normal distribution is discussed. The formulas are correct and the theory is presented with a clear language. The simulation study, although limited in its scope (see the comments below), is performed correctly and the results are well illustrated. The references are up to date and cover all the recent developments about the topic of the paper.

Although the paper contains an interesting piece of theory, there is room for improvements in order to reach the publication standards of PLOS-ONE.

Major comments.

I have basically only one major comment, concerning the simulation study. The authors present a simulation study for the expected value of a Normal distribution with known variance. This is a very strong limitation, since usually the normal distribution of an underlying process, as in the proposed application, has the two parameters both unknown. The authors claim at the end of the paper that the estimation of the variance and the extension to multi-dimensional parameters will be the subject of a subsequent paper, but I would suggest to add to the present paper some more scenarios in the simulation study, such as the mean **and** variance of a Normal distribution. Also the estimation of the expected value of other distributions, such as the exponential distribution should be considered.

Minor remarks.

1. Some sentences in the text must be rewritten. For instance, the first sentence of the introduction: "Bayesian inference deduces ..." should be "The aim of Bayesian inference is to deduce ...". Also in line 156, "becomes a tautology" is not logically correct.

2. Although numerical aspects are relevant in this paper, the use of 0.0 for 0 and 1.0 for 1 should be avoided.

3. Lines $135 - 140$. The meaning of the parameter m must be discussed.

4. Formula (10) in line 141 must be proved in full details. I do not see that it is an obvious consequence of the preceding formulas.

5. Line 205. The use of $\varepsilon = 10^{-300}$ must be checked with care. In several frameworks, such a value is too small for preventing from zero probabilities. I've tried in `R` the following:

   ```
   eps=10^{-300}
   a=1+eps
   b=a-1
   c=10^{300}*b
   ```

   obtaining

   ```
   > c
   [1] 0
   ```

6. Figure 1 can be removed.

7. The section "EM algorithm for Gaussian mixture learning" contains known material and can be considerably shortened.
