# Supplementary material for: A new method of Bayesian causal inference in non-stationary environments
Source: PLoS One. 2020 May 22;15(5):e0233559. doi: 10.1371/journal.pone.0233559 (PMC7244155; doi:10.1371/journal.pone.0233559)
Supplement: Attachment — Submitted filename: Response_to_Reviewers.docx [file pone.0233559.s002.docx]

Thank you for your careful peer review and helpful comments.

**Major comments.**

I have basically only one major comment, concerning the simulation study. The authors present a simulation study for the expected value of a Normal distribution with known variance. This is a very strong limitation, since usually the normal distribution of an underlying process, as in the proposed application, has the two parameters both unknown. The authors claim at the end of the paper that the estimation of the variance and the extension to multi-dimensional parameters will be the subject of a subsequent paper, but I would suggest to add to the present paper some more scenarios in the simulation study, such as the mean and variance of a Normal distribution. Also the estimation of the expected value of other distributions, such as the exponential distribution should be considered.

We have made the recommended changes to ensure that both the mean and variance can be estimated simultaneously. We have divided the section “Testing a normal distribution model” into two subsections, i.e., “Mean value estimation using normal distribution” and “Variance estimation using gamma distribution”; the latter describes the variance estimation. For the variance estimation, we have introduced a gamma distribution and found the solution using Lambert's W function. The results of the variance estimation are shown in Fig.6. With respect to this change, the whole manuscript has been modified to be consistent.

**Minor remarks.**

1. Some sentences in the text must be rewritten. For instance, the first sentence of the introduction: “Bayesian inference deduces…” should be “The aim of Bayesian inference is to deduce …”. Also in line 156, “becomes a tautology” is not logically correct.

Thank you for pointing this out.

We have added “The aim” and “to” in line 38.

“Bayesian inference deduces…”

🡪

“The aim of Bayesian inference is to deduce …”

We have modified line 170.

“…, formula (12) becomes a tautology as shown below:”

🡪

“…, $C^{t}\left(d^{t}|h_{k}\right)$ does not change by formula (14), as shown below.”

2. Although numerical aspects are relevant in this paper, the use of 0.0 for 0 and 1.0 for 1 should be avoided.

We modified 0.0, 1.0, and 5.0 in the text to 0, 1, and 5, respectively.

3. Lines 135-140. The meaning of the parameter *m* must be discussed.

Thank you for pointing out this important point.

We have interpreted your comment in two ways. One is that we thought you meant our explanation of parameter m was too brief. Therefore, we added lines 145-153 to clarify the meaning of parameter m. The other is that we thought you meant our treatment of the case where m = 0 was too brief, as you referred to lines 135-140. Therefore, we added lines 134-139 that contain the proof for the case m = 0.

4. Formula (10) in line 141 must be proved in full details. I do not see that it is an obvious consequence of the preceding formulas.

We have added the details for equation conversion in formula (12).

5. Line 205. The use of $\varepsilon$ = 10^-300^ must be checked with care. In several frameworks, such a value is too small for preventing from zero probabilities.

Thank you for pointing out this important point.

We have changed $\varepsilon$ = 10^-300^ to $\varepsilon$ = 10^-10^ in line 221.

6. Figure 1 can be removed.

According to this suggestion, we have removed Fig. 1.

7. The section “EM algorithm for Gaussian mixture learning” contains known material and can be considerably shortened.

Thank you for pointing this out.

We have deleted the section “EM algorithm for Gaussian mixture learning.” Moreover, we have included some necessary items in the section “Sequential Discounting Expectation-Maximisation Algorithm (SDEM).”
